# Changes in the dollar value of per capita alcohol, essential, and non-essential retail sales in Canada during COVID-19

**DOI:** 10.1186/s12889-021-12226-1

**Published:** 2021-11-25

**Authors:** Daniel T. Myran, Brendan T. Smith, Nathan Cantor, Lennon Li, Sudipta Saha, Catherine Paradis, Rebecca Jesseman, Peter Tanuseputro, Erin Hobin

**Affiliations:** 1 Clinical Epidemiology Program, Ottawa Hospital Research Institute, The Ottawa Hospital Civic Campus, 1053 Carling Ave, Ottawa, ON K1Y 4E9 Canada; 2 Public Health Ontario, Toronto, ON Canada; 3 Dalla School of Public Health, University of Toronto, Toronto, ON Canada; 4 Canadian Centre on Substance Abuse and Addiction, Ottawa, ON Canada; 5 Bruyère Research Institute, Ottawa, ON Canada; 6 Department of Medicine, University of Ottawa, Ottawa, ON Canada

## Abstract

**Background:**

Multiple survey reports suggest that alcohol use has increased in Canada during the COVID-19 pandemic. However, less is known about how per capita alcohol sales, which predict population-level alcohol use, have changed and whether changes in alcohol sales differ from changes in sales of other products due to pandemic factors.

**Methods:**

We obtained monthly retail sales data by industry from Statistics Canada, for the six largest provinces in Canada (containing 93% of the national population), between January 2010 and November 2020, representing time before and 9 months after the start of the pandemic in Canada. We used an interrupted time series analysis to estimate pandemic impacts on the dollar value of monthly per capita (per individuals 15+ years) alcohol, essential and non-essential retail sales. We adjusted our analyses for pre-pandemic sales trends, inflation, seasonality and changing population demographics over time.

**Results:**

During the first 9 months of the pandemic, the values of per capita alcohol, essential and non-essential sales were, respectively, 13.2% higher, 3.6% higher and 13.1% lower than the average values during the same period in the prior 3 years. Interrupted time series models showed significant level change for the value of monthly per capita alcohol sales (+$4.86, 95% CIs: 2.88, 6.83), essential sales (−$59.80, 95% CIs: − 78.47, − 41.03) and non-essential sales (−$308.70, 95% CIs: − $326.60, − 290.79) during the pandemic. Alcohol sales were consistently elevated during the pandemic, and the pre- and post-pandemic slopes were comparable. In contrast, essential and non-essential retail sales declined in the early months of the pandemic before returning to regular spending levels.

**Conclusion:**

During the first 9 months of the pandemic, per capita alcohol sales were moderately elevated in Canada. In contrast, non-essential sales were lower than prior years, driven by large decreases during the initial months of the pandemic. These findings suggest that the pandemic was associated with increased population-level alcohol consumption, which may lead to increased alcohol-related harms. Ongoing research is needed to examine how factors, including pandemic-related stressors and specific alcohol sales-related policies, may have influenced changes in alcohol use and harms.

**Supplementary Information:**

The online version contains supplementary material available at 10.1186/s12889-021-12226-1.

## Introduction

Alcohol is a leading cause of morbidity and mortality both in Canada and internationally [1, 2]. Since the start of the COVID-19 pandemic, there have been growing concerns about potential increases in alcohol use and consequent negative health impacts [3]. Alcohol use increases susceptibility to and severity of respiratory infections, which could facilitate the transmission and consequences of COVID-19 infections [4, 5]. In the short term increases in alcohol use could place an unnecessary burden on the acute health care system during a time of considerable strain [6]. In the longer term, alcohol’s contribution to multiple chronic diseases may mean that sustained increases in consumption could result in health and social costs well beyond the pandemic [2]. Multiple surveys have found increases in self-reported drinking during the pandemic. Results from two national surveys of Canadians found a 6 and 15% net increase in self-reported alcohol use in April and June 2020, respectively [7, 8]. However, surveys on alcohol use have conventionally been shown to dramatically underestimate alcohol consumption, primarily due to non-representative samples and inaccuracy of estimated self-reported alcohol use, and may not correctly capture pandemic-specific changes [9]. Consequently, there is ongoing uncertainty of the degree to which alcohol use has changed in Canada during the pandemic.

Per capita alcohol sales are an alternative to population-based surveys for monitoring changes in the population-level burden of alcohol. A number of studies have shown that increases in per capita alcohol sales are associated with greater alcohol consumption and harms [10]. A report from British Columbia estimated that the number of standard drinks consumed per capita increased by 7% during March – June 2020 compared to 2019 [11]. Another study from Ontario reported that the value of alcohol sold from the LCBO (a government agency responsible for the majority of alcohol sales in the province) during March – June in 2020 was 16.6% higher than in 2019 [12]. Alcohol sales have also increased in the United States (US) and United Kingdom (UK) during the pandemic. In the US, the mean volume of pure ethanol sold in all off- and on-premise retailers in 12 states in March, April and May 2020 were, respectively, + 2.9%, + 5.7% and − 1.0% compared to the same months in the prior 3 years [13]. Similarly, in the UK, early analyses found the overall value of alcohol sales increased in 2020 compared to previous years [14]. However, there has not been a comprehensive examination of how alcohol sales have changed across Canada during the pandemic [15]. Specifically, data are limited on how both “on” and “off” premise sales have changed (e.g. sales from both bars and restaurants and liquor stores), whether reported increases in alcohol sales during the early stages of the pandemic have continued over time, if sales are up after adjusting for inflation and growth trends over time and and how sales in regions other than British Columbia and Ontario have changed [16].

The pandemic has also caused substantial societal changes and disruptions resulting in large shifts in the type of products purchased by Canadians. In Canada, all 13 provinces and territories enacted periods of lockdown including the closure of non-essential business by March 21, 2020 until late April to mid-May 2020 [16]. During these periods of lockdown alcohol stores were designated as an essential service and allowed to remain open [17, 18]. While changes in alcohol sales are of immediate public health interest, comparing changes in alcohol to other retail sales can help isolate pandemic specific effects of alcohol consumption (e.g. did alcohol sales increase because of an overall trend of increased consumer spending or due to factors more specific to alcohol). In this study, we aimed to first understand if alcohol retail sales changed in Canada during the first 9 months of the pandemic. Then, we compared if and to what extent changes in alcohol retail sales differed from changes in sales of other goods and services designated as essential (e.g., groceries) and non-essential (e.g., clothing).

## Methods

### Study design and data sources

We used an interrupted time series (ITS) analysis to investigate if the dollar value of per capita alcohol sales in Canada changed during the first 9 months (March – November 2020) of the pandemic relative to prior years. We also examined changes in retail sales of essential and non-essential goods during the same time period. We used monthly sales data starting in January 2010 to November 2020 (latest available data at the time of analysis) from *Statistics Canada’s Monthly Retail Trade Survey (MRTS).* The MRTS estimates monthly sales (including “brick and mortar” and online) through repeated, cross-sectional, mandatory (response rate in 2018: 94.2%) surveys of retailers identified through the Canadian Business Registry [19]. We included sales from Quebec (QC), Ontario (ON), Manitoba (MB), Saskatchewan (SK), Alberta (AB) and British Columbia (BC), the six provinces for which Statistics Canada reports monthly alcohol sales. These provinces contain 93% of the Canadian population, and reported 93% of total alcohol retail sales (by value) for 2018/2019 [20].

We obtained the following data for provinces from Statistics Canada to calculate per capita sales and control for demographic confounders in our analyses; total population aged (15+), percent of population aged 20-29, and percent of population male [21]. We used assigned values from July 1st for each corresponding year of observation. We also obtained the monthly Consumer Price Index (CPI) for each jurisdiction from Statistics Canada to adjust for inflation and standardized all dollar values to January 2020 dollars [22].

### Primary outcome

Our primary outcomes were the inflation adjusted monthly dollar value (in CAD) of per capita retail sales (alcohol, and essential/non-essential goods) for individuals aged 15 + .

Retailers were classified into different industry groups according to the 4 digit-level North American Industry Classification System (NAICS 2017) [19]. Alcohol retail sales were estimated from retailers classified under “Beer, Wine and Liquor Stores” (NAICS code 4453 – a retailer whose primary business is selling packaged alcoholic beverages including beer, wine and liquor). The dollar value of monthly sales is estimated through a monthly sample of retailers (*n* = 1865 retailers on average per month in 2018) and sales reports from provincial liquor authorities. Off-premise alcohol sales from establishments that sell products other than alcohol, such as grocery stores and convenience stores, are only partially captured in the MRTS. Among jurisdictions in this study, alcohol is sold in convenience stores in Quebec and in select grocery stores in Ontario, Quebec, and British Columbia. Lastly, the MRTS indirectly captures on-premise (e.g. bars and restaurants) sales as provincial liquor authorities and sampled retailers report sales to establishments licensed to sell alcohol for on-premise consumption [23].

Prior to the COVID-19 pandemic, the majority of research on alcohol sales has used detailed data to estimate changes in the volume of pure ethanol purchased/consumed per capita over time. However, in this study we assessed changes in the per capita dollar value of alcohol sold. This data was used for two reasons: 1) examining the dollar value of alcohol sold facilitates comparisons to other sectors of Canada’s economy, including services deemed essential and non-essential during the pandemic and, 2) detailed alcohol sales data are not publicly available in many countries including Canada, and require special requests to individual provincial and territorial alcohol regulators. Several other reports have similarly used the value of alcohol sold to monitor changes in population-level use in a timely fashion during the pandemic [12, 14, 24].

We defined industry groups as “essential retail sales” if they were deemed integral to preserving life, health and basic social functioning and non-essential sales as all other industries. In effect, essential industries were permitted to continually operate with in-person customers during periods of lockdown while non-essential retail sales could only operate online or with curbside delivery during lockdowns. The essential and nonessential designations were determined using provincial policy documents [25]. The following industry groups were declared essential in all six provinces: grocery stores, convenience stores, automotive repair and maintenance, gas stations, health and personal care, building materials and garden stores and general merchandise stores. Non-essential industry groups in all provinces included: furniture sales, electronics and appliances, clothing and accessories, and sporting goods, hobby, book and music retailers [19]. New and used car sales were considered non-essential in all jurisdictions except Saskatchewan. Alcohol retailers were not included in the essential sales category total in our analyses.

### Statistical analyses

We calculated the inflation adjusted per capita (aged 15+) value of monthly sales (in dollars) for alcohol, essential and non-essential sales for each of the six provinces. We used the CPI to standardize sales to January 2020 using the following formula (monthly per capita sales * CPI score for month/ CPI score for January 2020). For alcohol sales we used the CPI values for on and off-premise alcohol retailers and for essential and non-essential sales we used the overall CPI values. We visualized the average retail sales from each of the six provinces to create a summary value for Canada. For each type of retail sales we calculated the relative percent change in the value of sales for the current year (March to November 2020) compared to the prior three-year average (March – November 2017 – 2019). We used segmented linear regression models to estimate the effect of the COVID-19 pandemic on changes in the value of alcohol, essential and non-essential sales. Our dependent variable for the three respective models was the monthly value of sales per capita (aged 15+). In order to generate stable time trends, we used all available sales data dating back to January 2010. The models included a binary pandemic indicator, (January 2010 – February 2020 compared to March 2020 – November 2020). The models included two continuous parameters for time; one for the time period before the pandemic and one for the time period during the pandemic. We included categorical variables for both month (12 categories) and province (6 categories) in the models to capture seasonality and differences among the provinces. We also included the percent of the population that is male and percent aged 20-29 as continuous covariates in convention with prior studies examining changes over time in alcohol sales [3]. Durbin–Watson tests for autocorrelation where significant (*p* < 0.001) for all three models so we used a first order monthly autoregressive structure.

We were primarily interested in three parameters in the model: the binary pandemic indicator, interpreted as the average monthly change in per capita spending during the pandemic; the pre- pandemic time parameter, interpreted as the monthly change in per capita sales before the pandemic; and the time during the pandemic parameter, interpreted as the monthly change in per capita sales during the pandemic. In order to understand how changes in the three outcomes (alcohol, essential and non-essential sales) differed, an additional regression model was fit with interaction terms between all variables in the model and the three outcomes. The interaction terms and the three key parameters were interpreted as the difference among outcomes. All tests of significance were two sided and we report 95% confidence intervals from the models. All analyses were conducted in R Version 4.2.

## Results

### Trends in per capita alcohol retail sales in Canada

**Fig. 1:**
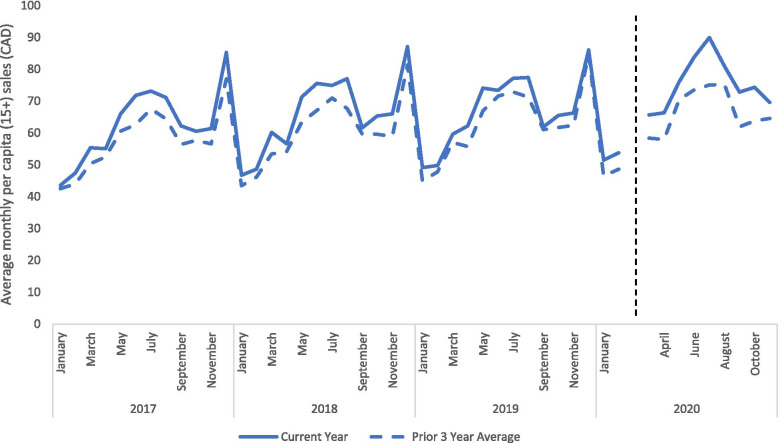
Per capita average monthly alcohol sales (in dollars per individual age 15+) comparing the most recent year to the 3 prior year average for the 6 largest jurisdictions in Canada. The dashed black line divides the pre- and during- COVID-19 period. The dollar value of sales are expressed in January 2020 constant dollars to adjust for inflation

Figure 1 presents the per capita value of alcohol sold in the six provinces in Canada during the pandemic compared to the average value of sales in the prior 3 years. Before the pandemic, alcohol sales exhibited a clear seasonal pattern, with the greatest sales in December, the lowest sales in January and February, increasing sales during March to August, and lower sales during September to November. During the pandemic, alcohol sales also had a similar seasonal pattern but the per capita value of alcohol sold during the first 9 months of the pandemic (March – November 2020) was 13.2% greater than the average value of alcohol sold during the same time period in in the prior 3 years (March – November, 2017-2019).

### Trends in per capita essential and non-essential retail sales in Canada

**Fig. 2:**
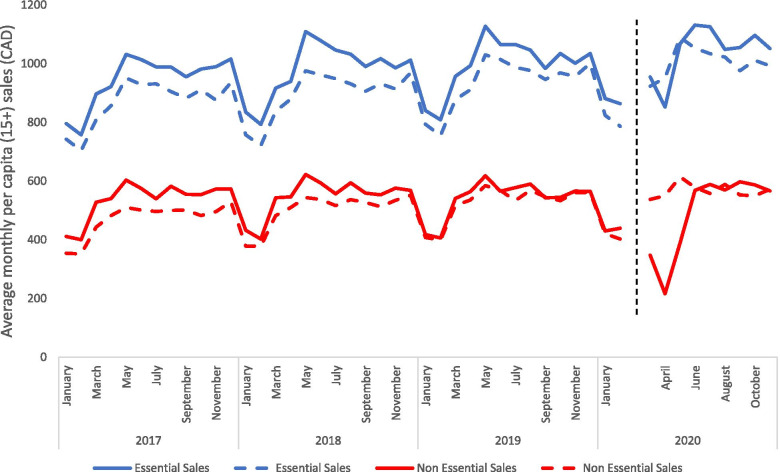
Per capita average monthly essential and non-essential retail sales (in dollars per individual age 15+) comparing the most recent year to the 3 prior year average for the 6 largest jurisdictions in Canada. The dashed black line divides the pre- and during- COVID-19 period. The dollar value of sales are expressed in January 2020 constant dollars to adjust for inflation

Figure 2 presents the per capita value of sales from essential and non-essential stores in the six provinces during the pandemic compared to the average sales of the prior 3 years. Essential and non-essential sales also show seasonal trends. Prior to the pandemic, essential and non-essential sales peaked during May and were at their lowest during January and February. The per capita values of essential and non-essential retail sales during the first 9 months of the COVID-19 pandemic (March – November 2020) were 3.6% greater and 13.1% lower, respectively, than the average values sold during the same time period in in the prior 3 years (March – November 2017-2019). During the first 3 months of the pandemic, the value of essential sales was lower than the prior 3-year average (− 3.0% decrease), and greater than the prior 3-year average during months 4 to 9 (+ 6.9% increase). The value of non-essential sales during the first 3 months of the pandemic showed a large decline compared to the prior 3-year average (44.2% decrease) and were relatively consistent with the prior 3-year average during months 4 to 9 (2.4% increase).

### Association of COVID-19 with changes in per capita retail sales

Table 1 presents the results of the ITS analysis on changes in alcohol, essential and non-essential sales for the six provinces using sales data between January 2010 and November 2020. After adjusting for inflation, all three types of sales showed a significant trend of increasing value of per capita monthly sales over time pre-pandemic. During the first 9 months of the pandemic, we observed a significant level increase in the monthly dollar value of per capita alcohol sales (+$4.86, 95% CIs: 2.88, 6.83) and significant level decreases in the monthly dollar value of per capita essential retail sales (−$59.80, 95% CIs: − 78.47, − 41.03) and non-essential retail sales (−$308.70, 95% CIs: -$326.60, − 290.79). Monthly alcohol sales were increasing at a similar rate pre-pandemic and during the first 9 months of the pandemic. In contrast, the rate of changes in monthly essential and non-essential sales in the first 9 months of the pandemic was greater compared to the pre-pandemic period, reflecting recovery from initial decreases in sales. The level change during the COVID-19 pandemic in alcohol sales was significantly different from the level change for essential (*p* = 0.006) and non-essential (*p* < 0.001) sales. The change in slope during the COVID-19 pandemic (secular trend per month) was also significantly different from the level change for essential (*p* < 0.001) and non-essential (*p* < 0.001) sales.

**Table 1:** Adjusted regression coefficients for absolute changes in the average monthly dollar value of per capita alcohol, cannabis, essential, and non-essential retail sales in Canada during the first 9 months of COVID-19 for the six largest provinces in Canada

Trend	Alcohol	Essential Sales	Non-Essential Sales
	Change in dollar (CAD)^**a**^ per capita aged 15+ (95% CI)^**b**^		
**Pre-COVID-19 slope** (secular trend per month)	+ 0.19 (0.18, 0.20) *p* < 0.001	+ 2.18 (210, 2.27) *p* < 0.001	+ 2.73 (2.65, 2.81) *p* < 0.001
**Level Change During COVID-19**^c^	+ 4.86 (2.88, 6.83) *p* = 0.02	− 59.8 (− 78.47, − 41.03) *p* = 0.002	− 308.70 (− 326.60, − 290.79) *p* < 0.001
**During COVID-19 slope** (secular trend per month)	+ 0.20 (− 0.14, 0.54) *p* = 0.56	+ 14.24 (11.00, 17.48) *p* < 0.001	+ 42.33 (39.23, 45.44) *p* < 0.001
**Percent Aged 20-29**	− 0.39 (−0.96, 0.18) *p* = 0.50	− 9.79 (− 14.99, − 4.60) *p* < 0.001	+ 73.11 (68.26, 77.97) *p* < 0.001
**Percent Male**	+ 1.61 (− 0.07, 3.28) *p* = 0.35	+ 82.06 (66.82, 97.30) *p* = 0.065	− 140.19 (− 154.44, − 125.96) *p* < 0.001

^a^The dollars value of sales are expressed in January 2020 constant dollars to adjust for inflation

^b^Changes in the dollar value of sales were adjusted for seasonal trends (categorical variable for each month) and for differences between the 6 provinces (categorial variable for each province)

^c^Reference = pre-pandemic period January 2010 – February 2020. Positive numbers indicate an increase in per capita sales, while negative numbers indicate declining sales

## Discussion

The present study is one of the first to date to comprehensively examine how alcohol sales have changed during the COVID-19 pandemic. We found that during the first 9 months of the pandemic (March to November 2020), the monthly value of per capita alcohol, essential and non-essential retail was respectively 13.2% higher, 3.6% higher and 13.1% lower than the prior 3-year average. Alcohol sales were consistently elevated compared to prior years. In contrast, the values of per capita essential and non-essential retail sales showed large decreases during the early months of the pandemic before returning to more normal levels of spending. Importantly, the per capita volume of ethanol sold is a well-established indicator of population-level alcohol consumption and an important predictor of alcohol-related harms [26]. We expect the increases in alcohol retail sales reported in our study will be reflected by similar increases in the volume sold and result in greater population-level alcohol harms in Canada in both the immediate and longer term.

Our results suggest Canadians have purchased and likely consumed moderately more alcohol than usual over the first 9 months of the pandemic. Importantly, we found that the surge in alcohol sales reported at the start of pandemic, thought to represent stockpiling behaviour, was not followed by lower sales in subsequent months [27, 28]. In addition, the sales data which captures sales from both on and off premise channels supports that despite well-documented decreases in sales from bars and restaurants, net sales of alcohol increased during the pandemic [29]. These findings are consistent with available regional and international sales data (UK and US) and alcohol industry reports [13–15].

There are a number of reasons which may explain why alcohol sales and use have increased in Canada and around the world. First, alcohol use may be a maladaptive coping strategy to manage the psychosocial distress from direct and indirect (e.g. job loss) impacts of the pandemic [3, 30]. Second, disruptions to routine and lack of regular schedule which previously constrained alcohol use may promote increased use [30]. Third, reduced access to alternative activities and commodities to alcohol may promote use. Finally, reduced access to preventive services, including reductions in primary care visits [31] and disrupted access to in-person supports, may have decreased access to services that reduce harmful alcohol use. In addition, during the pandemic many regulations and alcohol control policies were relaxed in Canada including allowing home delivery of alcohol from bars and restaurants, increasing the hours of sale from liquor stores, and reducing the price of some types of alcohol [32]. A large body of literature suggests that such changes likely contributed to increases in alcohol use and early reports from the alcohol industry cite new trends such as expanded online and direct to consumer sales as key to increases alcohol sales during this time period [26, 29].

It is important to contextualize changes in alcohol sales against general Canadian purchasing behavior. Overall spending on services deemed essential by the government was similar to prior years and Canadians spent far less money than usual on retail products designated as non-essential. While our data cannot determine the underlying cause of these consumer behaviours, the findings give greater support to the hypothesis that stressors and other factors related to the COVID-19 pandemic drove increases in alcohol sales. Importantly, problematic alcohol use results in an enormous health and health system burden in Canada and internationally [6, 33]. During the pandemic there was arguably a public health imperative to decrease alcohol use to reduce pressures on the health care system and protect individual mental health [2, 33]. Such a position was supported by the WHO, which recommended that alcohol control policies be maintained or strengthened during the COVID-19 pandemic [34]. Further research can help to better understand which factors may have contributed to both absolute increases in alcohol sales and relative increases compared to other sectors of the economy. Findings can inform policies during the remainder of the COVID-19 pandemic and during future public health emergencies.

### Strengths and limitations

Our study is one of the first in the literature to report changes in alcohol sales during the COVID-19 pandemic at the population-level. There are three main limitations of our study. First, changes in overall sales may overlook polarization, where some subgroups may increase substance use while others maintain or decrease their use. This possibility is supported by prior evidence from public health emergencies and surveys of Canadians over the course of the pandemic which found that individuals with poor self-reported mental health had greater increases in alcohol consumption than those with good self-reported mental health [35, 36]. Consequently, the moderate population-level changes in alcohol sales in our study may not capture increasing harms and inequities from alcohol in specific subgroups in Canada. Recent work has highlighted particular demographic groups that have experienced a disproportionate burden of emergency department visits due to alcohol in Ontario during the pandemic including those living in rural settings and lower-income neighbourhoods [37]. Second, while a robust body of literature has linked the volume of ethanol per capita sold to per capita consumption and harms, less research has examined the dollar value of alcohol sold. One limitation is that the increased dollar value of alcohol sold could reflect greater volume of sales, a switch to more expensive products, or a combination. Importantly, data from BC which estimated the volume of pure ethanol sold align with our findings [11]. In addition, while an imperfect measure, the value of alcohol sold has been used in several studies and reports to provide timely data on alcohol use during the pandemic [14]. Third, the data in our study only partially account for alcohol sales from supermarkets and convenience stores in Ontario and Quebec. We have likely underestimated changes in alcohol sales in these provinces as grocery stores across Canada reported a 40% increase in alcohol sales during March and April 2020 compared to 2019 [38]. Future work using complete alcohol sales to estimate the volume of alcohol per capita sold and consumed across Canada can determine if and to what extent this current study may have underestimated alcohol retail sales during the pandemic.

## Conclusion

Our findings suggest that per capita spending on alcohol use has moderately increased during the COVID-19 pandemic which will likely be reflected in greater population-level consumption of alcohol. In contrast, non-essential retail purchases showed large declines during the pandemic and spending on other essential goods was consistent with prior years. Increases in alcohol spending compared to maintained or decreased sales from other retail sectors suggest that pandemic related stressors and the associated governmental and societal response had an outsized influence on alcohol sales. Future research should examine factors that may have contributed to increased alcohol sales during the pandemic including specific policy decisions related to alcohol sales.

## Supplementary Information

**Additional file 1: Supplementary Table 1.** Classification of industries as essential or non-essential during COVID-19 for 12 Canadian jurisdictions. Shaded regions indicate essential industry designations.

## Data Availability

The data analysed during the current study are available from *Statistics Canada’s Monthly Retail Trade Survey* (https://www150.statcan.gc.ca/t1/tbl1/en/tv.action?pid=2010000802).
