# Supplementary material for: Changes in the dollar value of per capita alcohol, essential, and non-essential retail sales in Canada during COVID-19
Source: BMC Public Health. 2021 Nov 25;21:2162. doi: 10.1186/s12889-021-12226-1 (PMC8613522; doi:10.1186/s12889-021-12226-1)
Supplement: Supplementary file 1 — Additional file 1: Supplementary Table 1. Classification of industries as essential or non-essential during COVID-19 for 12 Canadian jurisdictions. Shaded regions indicate essential industry designations. [file 12889_2021_12226_MOESM1_ESM.docx]

**Supplementary Materials:**

**Additional file 1.**

Structured data extraction forms were used to identify essential services from publicly available policy documents. Essential services, defined at the provincial/territorial level, are services and functions deemed essential to preserving life, health and basic social functioning, for example, first responders, health care, critical infrastructure (e.g., hydro), and critical goods (e.g., food and medicine). Essential services listed for each province/territory in Canada were manually mapped onto industries using 4-digit North American Industry Classification System (NAICS) codes (lowest level) to classify essential and non-essential services.

During the months of March, April and May the following industries were declared essential in all jurisdictions in Canada; automatic parts, accessories and tire stores (4413), building material and garden stores (444), supermarkets and grocery stores (44511), convenience stores (44512), specialty food stores (4452), health and personal care stores (446), gasoline stations (447), and general merchandise stores (452). The following industries were declared non-essential in all jurisdictions in Canada; furniture and home furnishing stores (4421-4422), electronics and appliance stores (443), clothing and accessories stores (448), and sporting goods, hobby, book and music stores (451). Automotive dealers (4411) and other vehicle dealers (4412) were declared essential in five jurisdictions (North West Territories, Saskatchewan, New Brunswick, Nova Scotia, and Newfoundland) and non-essential in the remaining 7 jurisdictions.

**Supplementary Table 1. Classification of industries as essential or non-essential during COVID-19 for 12 Canadian jurisdictions. Shaded regions indicate essential industry designations.**

|  | **Jurisdiction** | **ON** | **ALTA** | **BC** | **QC** | **SK** | **MB** | **NFLD** | **PEI** | **NS** | **NB** | **Yukon** | **NWT** |
| --- | --- | --- | --- | --- | --- | --- | --- | --- | --- | --- | --- | --- | --- |
| **Industry** | **2017 NAICS CODE** | **Industry Designated as Essential in Jurisdiction** | | | | | | | | | | | |
| **Motor vehicle (4411-4413),** | 4411-4413 | - | - | - | - | - | - | - | - | - | - | - | - |
| **Automobile dealers** | 4411 | No | No | No | No | Yes | No | Yes | No | Yes | Yes | No | Yes |
| **Other motor vehicle dealers** | 4412 | No | No | No | No | Yes | No | Yes | No | Yes | Yes | No | Yes |
| **Automotive parts, accessories, and tire stores** | 4413 | Yes | Yes | Yes | Yes | Yes | Yes | Yes | Yes | Yes | Yes | Yes | Yes |
| **Furniture and home furnishing stores** | 4421-4422 | No | No | No | No | No | No | No | No | No | No | No | No |
| **Electronics and appliance stores** | 443 | No | No | No | No | No | No | No | No | No | No | No | No |
| **Building material and garden stores** | 444 | Yes | Yes | Yes | Yes | Yes | Yes | Yes | Yes | Yes | Yes | Yes | Yes |
| **Supermarkets and grocery stores (44511),** | 44511 | Yes | Yes | Yes | Yes | Yes | Yes | Yes | Yes | Yes | Yes | Yes | Yes |
| **Convenience stores (44512),** | 44512 | Yes | Yes | Yes | Yes | Yes | Yes | Yes | Yes | Yes | Yes | Yes | Yes |
| **Specialty food stores (4452** | 4452 | Yes | Yes | Yes | Yes | Yes | Yes | Yes | Yes | Yes | Yes | Yes | Yes |
| **Health and personal care stores (446** | 446 | Yes | Yes | Yes | Yes | Yes | Yes | Yes | Yes | Yes | Yes | Yes | Yes |
| **Gasoline stations (447)** | 447 | Yes | Yes | Yes | Yes | Yes | Yes | Yes | Yes | Yes | Yes | Yes | Yes |
| **Clothing and accessories stores (448** | 448 | No | No | No | No | No | No | No | No | No | No | No | No |
| **Sporting goods, hobby, book, and music stores** | 451 | No | No | No | No | No | No | No | No | No | No | No | No |
| **General merchandise stores** | 452 | Yes | Yes | Yes | Yes | Yes | Yes | Yes | Yes | Yes | Yes | Yes | Yes |
